# Double Pituitary Incidentaloma in a Young Woman With Sinusitis-Related Headache

**DOI:** 10.1210/jcemcr/luad170

**Published:** 2024-01-04

**Authors:** Pedro Marques, Lia Neto, Inês Sapinho, Amets Sagarribay

**Affiliations:** Endocrinology Department, Pituitary Tumor Unit, Hospital CUF Descobertas, Lisbon 1998-018, Portugal; Faculty of Medicine, Universidade Católica Portuguesa, Rio de Mouro, Lisbon 2635-631, Portugal; Radiology Department, Pituitary Tumor Unit, Hospital CUF Descobertas, Lisbon 1998-018, Portugal; Endocrinology Department, Pituitary Tumor Unit, Hospital CUF Descobertas, Lisbon 1998-018, Portugal; Neurosurgery Department, Pituitary Tumor Unit, Hospital CUF Descobertas, Lisbon 1998-018, Portugal

**Keywords:** pituitary incidentaloma, pituitary adenoma, Rathke cleft cyst, sinusitis

## Image Legend

A 21-year-old-woman presented with a 1-month history of headaches. Head computed tomography (CT) and magnetic resonance imaging (MRI) revealed acute sphenoid sinusitis (*, panels E and F), likely causing her headaches. Gadolinium-enhanced MRI also showed a right 7-mm pituitary lesion (arrowheads) with poor contrast enhancement on T1-weighted images (panels A and D), presumed as a pituitary adenoma (PA), and a separate 8-mm lesion anterior to the stalk (arrows) with low-intensity on T1 (panels A and D) and high-intensity on T2-weighted images with an intracystic nodule (panels B and C), compatible with Rathke cleft cyst (RCC). She had regular menstrual cycles, no polydipsia or polyuria, and her pituitary function and serum prolactin were normal. Headaches resolved following sinusitis-directed treatment. RCCs can be found in 13% to 22% of normal pituitaries, but rarely coexist with PAs. Concomitant RCC and PA often occurs on MRI evaluation for PA patients, or following surgery for a PA [1, 2]. However, it is unusual to encounter a RCC and a presumed nonfunctioning PA incidentally found in a patient investigated for headaches, none causing this presenting manifestation. This case also alerts for the need of considering other etiologies for headaches in patients with small RCCs and/or nonfunctioning PAs to prevent inadequate management.

**Figure:**
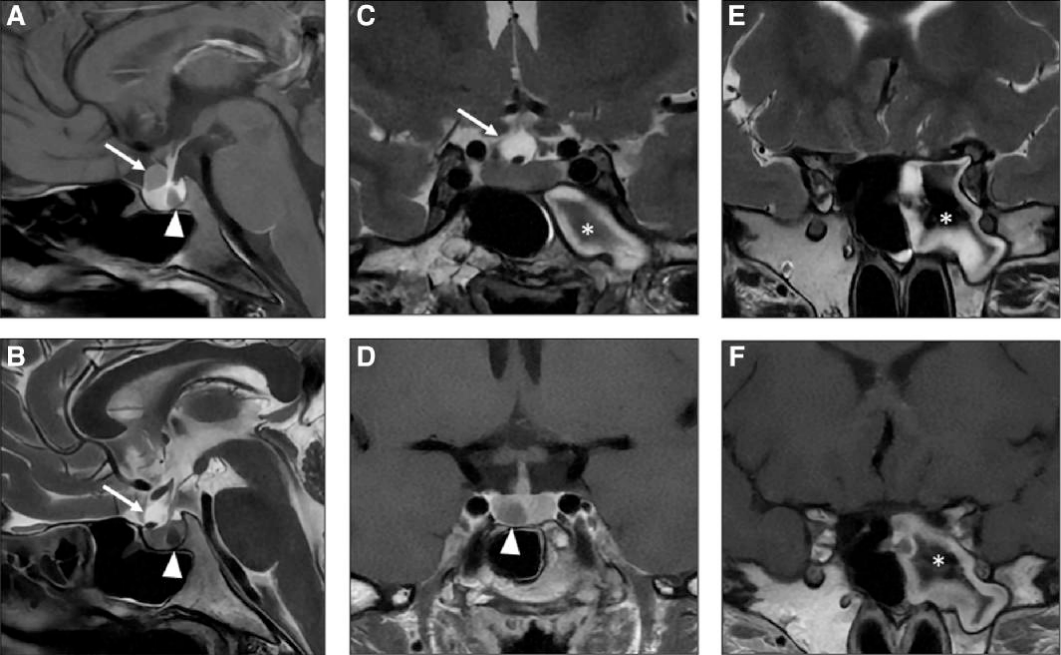


## Data Availability

Original data generated and analyzed for this case report are included in this published article.

## Abbreviations

CT: computed tomography
MRI: magnetic resonance imaging
PA: pituitary adenoma
RCC: Rathke cleft cyst
